# Risk factors for mortality in fournier's gangrene in a general hospital: use of simplified founier gangrene severe index score (SFGSI)

**DOI:** 10.1590/S1677-5538.IBJU.2017.0193

**Published:** 2018

**Authors:** Carlos Eugênio Lira Tenório, Salvador Vilar Correia Lima, Amanda Vasconcelos de Albuquerque, Mariana Pauferro Cavalcanti, Flávio Teles

**Affiliations:** 1 Serviço de Urologia do Hospital das Clínicas, Departamento de Cirurgia do Centro de Ciências da Saúde da Universidade Federal de Pernambuco, UFPE, Recife, PE, Brasil; 2 Faculdade de Medicina da Universidade Estadual de Ciências da Saúde de Alagoas (UNCISAL), Maceió, AL, Brasil; 3 Núcleo de Cirurgia Experimental, Programa de Pós-Graduação em Cirurgia, Departamento de Cirurgia, Centro de Ciências da Saúde, Universidade Federal de Pernambuco, UFPE, Recife, PE, Brasil

**Keywords:** Fournier Gangrene, Risk Factors, Mortality, Fasciitis, Necrotizing

## Abstract

**Objective:**

To evaluate risk factors for mortality in patients with Fournier's gangrene (FG), with emphasis in the Simplified Fournier Gangrene Severe Index Score (SFGSI).

**Materials and Methods:**

This was a cross-sectional study that was carried out from January 2010 to December 2014, with 124 patients treated for FG in a General Hospital. Several clinical and laboratory variables, including SFGSI, were evaluated and correlated with mortality through univariate analysis and logistic regression.

**Results:**

Of the 124 patients, 99 were men (79.8%), the mean age was 50.8±19.5 years and the main comorbidity was diabetes mellitus (51.6%). The mortality rate was 25.8%. Variables that presented independent correlation with mortality were the extension of the lesion to the abdomen (OR=4.0, CI=1.10-14.68, p=0.03), hematocrit (OR=0.81, CI=0.73-0.90, p<0.0001), potassium (OR=2.41, CI=1.13-5.10, p=0.02) and creatinine (OR=2.15, CI= 1.04-4.41, p=0.03). When hematocrit, potassium and creatinine were tested together, as part of the SFGSI, a >2 result was the largest of the independent predictors of mortality (OR=50.2; CI=13.18-191.47; p<0.0001).

**Conclusion:**

The SFGSI >2 presented a higher correlation with mortality than any variable tested alone. It seems to be a promising alternative to evaluate predictors of mortality in Fournier's gangrene. The main advantage is easy applicability because it contains only three parameters and can be used immediately after patient's admission.

## INTRODUCTION

Fournier's gangrene (FG) is a potentially fatal disease characterized by necrotizing fasciitis of the perineal and genital region resulting from synergistic polymicrobial infection (1-5). It affects mainly males, between the third and sixth decade of life, but it may be found in all age groups, even in women and newborns. In the US, the incidence is 1.6/100.000 men, with peak age between 50 and 79 years (6). The etiology of infection may be idiopathic, or secondary to anorectal, urogenital and cutaneous diseases, among others such as trauma and prior surgical procedures (7). The main predisposing factors associated with FG are: diabetes; alcoholism; hypertension and smoking, both predisposing to obliterating endoarteritis; and immunosuppressive diseases such as malignant neoplasms and HIV (2, 8).

Despite the broadly diverse microorganisms in the etiology, its treatment is unique in all cases, including emergency surgical removal of devitalized tissues, intravenous administration of broad-spectrum antibiotics, and rigorous clinical and hemodynamic support (9-13). Nevertheless, FG is still a disease with a high mortality rate (14). Mortality rates are between 20 and 40% in most cases, but they range from 4 to 88% (6).

In the last two decades, several studies have described the use of different scoring systems in predicting the mortality of patients with Fournier's gangrene (15-17): the Fournier Gangrene Severe Index Score (FGSI), which included nine clinical and laboratory parameters (15); Uludag Fournier Gangrene Severe Index Score (UFGSI), which added age and extent of the disease to the FGSI (10); age-adjusted Charlson Comorbidity Index (ACCI), in which 19 comorbidities were recorded, with an additional score added for age (16); and the Surgical Apgar Score (sAPGAR), in which blood loss, lower mean blood pressure and lower heart rates are considered and scored during surgery (17).

In clinical practice, a simplified and reliable scoring system would be easily accepted by the medical and academic community. In this direction, Lin et al. developed a reduced score in 2014, named the simplified FGSI (SFGSI), with only 3 variables, with the advantage of use only few parameters, without losing its sensitivity and specificity (18). The objective of the present study was to demonstrate the main risk factors for mortality in patients with FG in a general hospital, with emphasis on SFGSI.

## MATERIALS AND METHODS

The project was approved by the local Ethical Committee of the Institution and registered in Plataforma Brasil under number 46897115.2.0000.5011. Data from patients submitted to emergency surgery for FG were extracted from the medical charts of patients admitted to the General Public Hospital, from January 2010 to December 2014. The diagnosis was based on the clinical history, and signs and symptoms such as, fever, erythema, edema, fluctuation, crepitation and necrosis in the perianal, perineal and or genital areas. Patients with perianal, periurethral and scrotal abscesses in which there was no evidence of necrosis and extension to soft tissues were excluded from the analysis. Surgeries were performed by the urology team of the referred hospital.

Clinical data such as age, sex, length of hospital stay, major risk factors, areas of involvement, extent of infection, etiology, isolated microorganism, cystostomy and or colostomy, and mortality rate were analyzed. Laboratory data collected at admission, such as plasma glucose, hematocrit (Hct), potassium (K) and creatinine (Cr) were also analyzed. Through the result of Htc, K and Cr, the simplified FGSI (SFGSI) was applied, which consists of a punctuation system with the three variables mentioned above. In this score, values from 0 to 4 are attributed for each variable and the SFGSI is calculated by adding the points of each parameter. The cutoff point is two, and a value higher than 2 has a sensitivity of 87% and specificity of 77% to predict mortality (18).

All patients underwent at least one radical debridement of the affected devitalized tissues. A colostomy was performed when there was a perirectal infection with involvement of the anal sphincter, in conjunction with the general surgery team. A suprapubic cystostomy was performed in cases of periurethral origin with evidence of urinary extravasation. Antibiotic therapy with ciprofloxacin and metronidazole was initiated in the emergency room and maintained during hospitalization and was only modified, according to the result of the microbiological analysis of the tissue samples removed at the first debridement and under the guidance of the local hospital infection commission. Patients underwent additional debridement, when necessary, during wound explorations. The debridement continued until the removal of all devitalized tissues and the establishment of healthy granulation tissue on the wound. Most of the patients were treated in wards. During hospitalization, patients with severe sepsis, who required mechanical ventilation or hemodynamic support, were treated at the intensive care unit (ICU).

Numerical variables were expressed as mean±standard deviation (SD) or median with interquartile range, after the Kolmogorov-Smirnov normality test. The associations among the continuous variables were performed by Student's t-test and chi-square test for categorical variables. Some variables were compared according to mortality. The variables that correlated with mortality in the univariate analysis had their risk adjusted by logistic regression. A significance level of p <0.05 and a confidence interval of 95% were adopted. The whole statistical analysis was performed using the statistical program Statistical Package for the Social Sciences (SPSS version 20).

## RESULTS

The main clinical and laboratory data are shown in Table-1. One hundred and twenty-four patients were included in the study, 79.8% of which were men. The average age was 50.8±19.5 years. The mean time of hospitalization was 21.7±26.8 days. Among the well stablished risk factors for FG, the study identified diabetes mellitus in 64 patients (51.6%), hypertension in 32 cases (25.8%), chronic alcoholism in 27 (21.7%), immunosuppressive drugs (use of glucocorticoids and post-chemotherapy) in 21 (16.9%) and smoking in 19 (15.3%) cases. In regard to the sites affected, the genitalia was compromised in 106 cases (85.48%), the perineal region in 78 (62.9%), and the perianal region in 64 (51.61%). The extent of infection to the abdominal wall occurred in 44 patients (35.48%). In addition to surgical debridement of devitalized tissues, colostomy was required in 15 cases (12.1%), cystostomy in 15 (12.1%), unilateral orchiectomy in 9 (7.2%) and plastic reconstruction in 14 (11.3%). The hyperbaric chamber was used in only 1 patient (0.8%). Regarding the microorganisms found in qualitative cultures of debrided tissues, the majority was classified as “mixed flora”. The main microorganisms found were: Gram negative bacteria (Escherichia coli, Proteus mirabilis, Klebsiella sp, Pseudomonas), Gram positive bacteria (Staphylococci, Enterococcus, Clostridium) and less commonly fungi.

**Table 1:** Main Clinical and laboratory data (n=124).

Variables	Results
Age (years)	50.8±19.5
Male	99 (79.8%)
Length of hospital stay (days)	21.7±26.8
Diabetes mellitus	64 (51.6%)
Hypertension	32 (25.8%)
Extension to the abdomen	44 (35.5%)
Colostomy	15 (12.1%)
Cistostomy	15 (12.1%)
Orchiectomy	9 (7.3%)
Hematocrit (%)	31.7±6.7
Sodium (mmol/L) (n=53)	138±10.8
Potassium (mmol/L)	4±0.7
Creatinine (mg/dL)	1.5±0.82
Glucose (mg/dL) (n=64)	212±126
SFGSI>2	40 (32.3%)
Death	32 (25.8%)

Results expressed as mean ± standard deviation or absolute number and percentage.

The overall mortality of the sample was 25.8%. The SFGSI was applied in all patients at the admission and 40 (32.2%) of which had a result >2. The mortality of patients with SFGSI >2 was of 70%, versus 4.8% of patients with SFGSI <2; p<0.0001.

The main variables were also analyzed in relation to the occurrence of death (Table-2). Among the non-survivors there was a higher mean age (58.47±20.14, versus 48.15±18.66 in survivors; p=0.01), lower hematocrit (26.4±5.1, versus 33.5±6.3 in survivors; p <0.0001), higher mean of potassium (4.45±1.09, versus 3.84±0.5 in survivors; p=0.004) and creatinine (1.72±1.16, versus 0.95±0.55 in survivors; p<0.001) and higher mean of SFGSI (4.78±1.66, versus 0.97±1.58 in survivors; p<0.0001). Diabetic patients had a higher mortality when compared to non-diabetic patients (71.9% versus 44.6%; p=0.01), and the extension of the disease to the abdomen was also more frequent among non-survivors (53.1% versus 29.3%; p=0.01). The remained variables showed no significant differences regarding mortality in univariate analysis.

**Table 2:** Distribution of variables according to mortality.

Variables	Survivors (n = 92)	Non Survivors (n = 32)	p
Age (years)	48.15±18.66	58.47±20.14	0.01
Male	76 (82.6%)	23 (71.9%)	0.20
Length of hospital stay (days)	22.14±26.4	20.72±28.3	0.80
Diabetes mellitus	41 (44.6%)	23 (71.9%)	0.01
Hypertension	24 (26.1%)	8 (25%)	1
Extension to the abdomen	27 (29.3%)	17 (53.1%)	0.01
Colostomy	8 (53.3%)	7 (46.7%)	0.1
Cistostomy	10 (10.9%)	5 (15.6%)	0.53
Orchiectomy	7 (7.6%)	2 (6.3%)	1
Hematocrit (%)	33.5±6.3	26.4±5.1	0.0001
Sodium (mmol/L) (n=53)	136±5.7	140±14.7	0.19
Potassium (mmol/L)	3.84±0.5	4.45±1.0	0.004
Creatinine (mg/dL)	0.95±0.55	1.72±1.16	0.001
Glucose (mg/dL) (n=64)	210±122.4	215±135.9	0.87
SGFSI>2	0.97±1.58	4.78 ± 1.66	0.0001

Results expressed as mean ± standard deviation or absolute number and percentage.

**SFGSI** = Simplified Fournier Gangrene Severe Index Score.

Logistic regression was applied to evaluate risk factors for death (Table-3). Variables that correlated independently with death were the extension of the lesion to the abdomen (OR=4.0, CI= 1.10-14.68; p=0.03), hematocrit (OR=0.81, CI=0.73-0.90; p<0.0001), potassium (OR=2.41, CI=1.13-5.10; p=0.02) and creatinine (OR=2.15, CI=1.04-4.41; p=0.03). When hematocrit, potassium and creatinine were tested together, as part of the SFGSI, a value >2 was the largest independent predictor of mortality (OR=50.2; CI=13.18-191.47; p<0.0001). The remained variables (age and diabetes) showed no correlation with death in logistic regression.

**Table 3:** Independent risk factors for mortality (logistic regression).

Variable	OR	CI	p
Age	1.02	0.99-1.06	0.13
Diabetes mellitus	2.4	0.68-8.39	0.17
Extension to the abdomen	4	1.10-14.68	0.03
Hematocrit	0.81	0.73-0.90	<0.0001
Potassium	2.41	1.13-5.10	0.02
Creatinine	2.15	1.04-4.41	0.03
SFGSI>2	50.2	13.18-191.47	<0.0001

**OR =** odds ratio; **CI =** confidence interval; **SFGSI =** Simplified Fournier Gangrene Severe Index Score.

## DISCUSSION

Among the major scoring systems for predicting mortality in Fournier gangrene, the FGSI remains the most used with a sensitivity of 6588% and specificity of 70-100% (15, 19-22). The FGSI consists of nine clinical and laboratory parameters (temperature, heart rate, respiratory rate, sodium, potassium, creatinine, leukocytes, hematocrit and bicarbonate). In this index, for each parameter a score from 0 to 4 is assigned and the FGSI is calculated by adding the points of each parameter. The cut-off point is nine, meaning that when FGSI is >9, the probability of death is 75%, and when it is <9, the probability of survival is 78% (15). Although FGSI has demonstrated its role in predicting mortality, a thorough review of the literature has shown that not all FGSI parameters were significantly different between survivors and non-survivors (20-22).

The major disadvantage of FGSI and other scores such as the UFGSI is that they are difficult to apply at patient admission because they include many variables (15, 10). In daily practice, a more simplified scoring system, consisting of fewer variables would be more useful. In a recent study, Lin et al. demonstrated that plasmatic hematocrit, creatinine and potassium are the FGSI variables that most correlate with mortality. In this study, the use of the three variables together, forming a score, was not inferior to the FGSI and showed a sensitivity of 87% and specificity of 77%, when the sum of the score was greater than 2 (18). In this study, we verified that the simplified form of FGSI (SFGSI) was highly predictive of mortality in our sample. The strong point of the present study is that it demonstrates the applicability of SFGSI in the management of patients with FG. To the best of our knowledge, this is the largest study that evaluates the use of SFGSI as a predictor of mortality in FG.

Hematocrit, creatinine and potassium, associated with other clinical and laboratory parameters may be prognostic factors related to FG mortality. In the study carried out by Erol et al. on 18 patients with FG, individual laboratory parameters such as hypomagnesemia, hemoglobin and hematocrit reduction, and creatinine, urea and potassium increase were associated with worse prognosis, as was the FGSI >9 (23). In a recent article, Garcia Marin et al. evaluated several clinical and laboratory parameters of 59 FG patients, in addition to FGSI and UFGSI. In the multivariate analysis, the main parameters associated with mortality were peripheral vasculopathy, serum potassium and severe sepsis (24). In the study by Lin E et al., low hematocrit, low albumin and high FGSI were the factors associated with high mortality (21).

It was also observed in our study that hematocrit, creatinine and potassium were independent risk factors for mortality. However, when we tested these 3 variables together (SFGSI) there was a significant increase in relative risk. The advantage of SFGSI is the use of only 3 laboratory variables, which are easy to quantify, making it a score with high applicability potential in the admission and follow-up of FG patients.

We also verified that the extension of the lesion to the abdominal wall was a predictor of mortality. There is no consensus whether the extent of FG infection is associated with a poorer prognosis. Spinark et al. reported high mortality rate in patients with a large impaired area (25). This finding was also confirmed by other authors (26-28). Other series do not demonstrate a direct relationship between a large impaired area and prognosis, probably because a reduced patient sample (15, 19, 20).

This study has some limitations, which have to be pointed out. We recruited patients over a period of 5 years, and potential bias might exist including data quality and different clinical practices. As this was a retrospective study and performed in a single center we cannot generalize our findings to other populations.

We believe that the simplified FGSI contains minimal parameters, it is fast, easy to use at initial diagnosis, it can facilitate risk stratification of Fournier's gangrene and detect patients who are at high risk of mortality. However, more studies are needed to validate its application in different populations.

## CONCLUSIONS

FG still has a high mortality rate. The extent of the lesion to the abdomen, plasmatic levels of hematocrit, potassium and creatinine are independent risk factors for mortality. The simplified form of FGSI showed a higher correlation with mortality than any variable tested alone. It seems to be a promising alternative to evaluate predictors of mortality in Fournier's gangrene. The main advantage is easy applicability because it contains only three parameters and can be used immediately after patient's admission.
